# Sex-modified association between grip strength and mild cognitive impairment: a cross-sectional and follow-up study in rural China

**DOI:** 10.1186/s12877-023-04376-1

**Published:** 2023-11-02

**Authors:** Wenjing Feng, Qian Chen, Mingfeng Ma, Jiahui Xu, Hui Guo, Wei Yuan, Ruixue Li, Hanshu Gao, Cuiying Gu, Yanan Ma, Zhaoqing Sun, Nan Tuo, Liqiang Zheng

**Affiliations:** 1 The International Peace Maternity and Child Health Hospital, Shanghai Jiao Tong University School of Medicine, Shanghai, China; 2 Department of Biostatistics and Epidemiology, School of Public Health, China Medical University, Shenyang, China; 3 Department of Neurology, Shengjing Hospital of China Medical University, Shenyang, China; 4 Department of Cardiology, Fenyang Hospital of Shanxi Province, Fenyang, Shanxi China; 5 Institute of Health Sciences, China Medical University, Shenyang, China; 6 Department of Cardiology, Shengjing Hospital of China Medical University, Shenyang, China

**Keywords:** Handgrip strength, Mild cognitive impairment, Cross-sectional study, Cohort study, Sex

## Abstract

**Background:**

The sex difference in the association between grip strength and mild cognitive impairment (MCI) remains controversial and unclear.

**Methods:**

This is a part of a chronic disease cohort study conducted in rural areas, Fuxin, Liaoning Province, China. At the baseline survey, a total of 2633 participants aged 35- 85 were included in the cross-sectional study. Handgrip strength (HGS, kg) was measured by a dynamometer (Jamar +). MCI were assessed using the Chinese version of the Montreal Cognitive Assessment-Basic (MOCA-BC). Then, a total of 1667 cognitively normal individuals (NCs) were planed to follow up and to assess the incident MCI after two years. We used logistic regression to examine the association between HGS (as a continuous variable and quintiles) and MCI and analyzed the interaction between sex and HGS on MCI. Models stratified by sex were adjusted for demographic information (age, ethnicity, education, marital status, income, physical labor level), modifiable risk factors (body mass index, smoking, drinking) and disease history (hypertension, diabetes, dyslipidemia and coronary heart disease). Baseline MOCA-BC scores were additionally adjusted in the longitudinal study.

**Results:**

In the cross-sectional study, participants were on average 56.6 ± 9.8 years, and 1713 (65.1%) were females. In the cohort study, 743 individuals were followed up with an average age of 55.9 ± 9.6 years, which included 530 (71.3%) females. The cumulative incidence of MCI over a two-year period was 17.1%. In the cross-sectional study, compared to the highest quintile of HGS, the lowest HGS was associated with higher risk of MCI in males (odds ratio [OR]: 2.66; 95% confidence interval [CI]: 1.54, 4.64) and females (OR: 1.70; 95% CI: 1.17, 2.49) with adjustment of potential confounding factors. In the cohort study, compared to the highest quintile of HGS, the lowest HGS was associated with an increased risk of incident MCI in females (OR: 3.93; 95% CI: 1.39, 13.01) but not in males (OR: 0.56; 95% CI: 0.11, 2.94, *P* _for interaction_ = 0.015).

**Conclusions:**

Lower grip strength is a risk factor for mild cognitive impairment and predicts a higher risk of MCI in females.

**Supplementary Information:**

The online version contains supplementary material available at 10.1186/s12877-023-04376-1.

## Background

According to the 2021 Global Status Report released by the WHO, disability-adjusted life years (DALYs) caused by dementia have increased by 122% over the past 20 years, which is the most important reason among the top 30 causes of DALYs [1]. As there is no effective treatment for dementia, mild cognitive impairment (MCI), which is considered a transitional phase between normal cognitive aging and Alzheimer's disease (AD), has been increasingly emphasized. Studies have shown that individuals with MCI have an increased risk of incident dementia compared with cognitively normal individuals (NCs) [2]. Furthermore, a study found that women had a higher risk of dementia than men [3, 4], and this was also found in pathological changes [5].

Previous studies showed that dementia and MCI patients had a decline in physiological functions, such as lower muscle mass, slower gait and lower muscle strength [6, 7]. The relationship and mechanism between these changes and cognition will provide clues to the etiology and early markers of dementia. A recent study showed that muscle mass did not predict late-life cognitive impairment, while muscle strength mediated this association [8]. Gait speed, which has also been shown to predict cognitive decline [9], may be limited by certain health limitations (e.g., physical disabilities) and difficult to monitor in health care in low development areas (e.g., test environment). However, muscle strength can be assessed by handgrip strength (HGS), which is stable, reliable, convenient and low cost [10]. So, we believe that the study on the relationship between grip strength and cognitive impairment is more suitable for promotion and application in rural areas.

Many studies have shown a correlation between HGS and cognitive performance, and a previous scoping review concluded that HGS can be used to monitor individual cognitive decline [11]. However, prospective studies showed mixed results. Some studies found that lower HGS at baseline was associated with a higher risk of incident MCI [8, 12, 13]. In contrast, a recent study from a cohort of older adults in Mexico showed no longitudinal association between HGS and subsequent MCI [14], and a Korean study reached similar conclusions [15]. Only a few studies have directly investigated sex differences in the association between HGS and cognitive impairment. Female grip strength is lower than male in the general population, and studies have shown that female have better cognitive reserve than male, especially language and memory [16]. Under the same pathological damage, women's cognitive performance is stronger, but once it is impaired, its cognitive performance will decline faster than men [17, 18]. Therefore, the relationship between grip strength and age-independent cognitive decline may be inconsistent across sex.

We hypothesized that sex modified this association, and studying this sex difference could lead to the better use of grip strength as a predictor in clinical and public health strategies. Therefore, we conducted a longitudinal study from rural areas in China to explore the association between HGS and MCI using both cross-sectional and longitudinal designs to provide more specific evidence. We sought to assess the association of HGS with cognitive impairment and risk of future incident MCI in males and females, respectively, and the moderating effect of sex on the association between HGS and cognitive impairment.

## Methods

### Study population

This study was a part of a National key research and development program in China, which aimed to provide scientific basis for the prevention and control of chronic diseases among middle-aged and elderly people (≥ 35 years) in northeast China. The cohort study was conducted in rural areas of Fuxin Mongolia Autonomous County, Liaoning Province, China. The baseline survey was conducted in 2019, which included standardized procedures for resident questionnaire survey, health examination, and collection of blood samples from participants. A comprehensive follow-up was planned every 2 years, including health examination and collection of biological samples. Samples were collected from 33 villages of 4 townships according to their demographic characteristics (Han and minority areas) and geographical location (east, south and north of this region). Participants were eligible if (1) they were aged ≥ 35; (2) they had lived in this area for at least five years; and (3) they were willing to provide written informed consent prior to enrollment and excluded if (1) they were pregnant; (2) they developed severe liver and renal failure; and (3) they were unwilling to participate in this study. Initially, 4689 participants were recruited and investigated as the baseline population. From March 2021—June 2021, the second wave of the survey was conducted. The data were collected using standardized questionnaires and recorded by trained investigators. Written informed consent was obtained from all participants. This study was approved by the human experimentation committee of China Medical University.

**Fig. 1:**
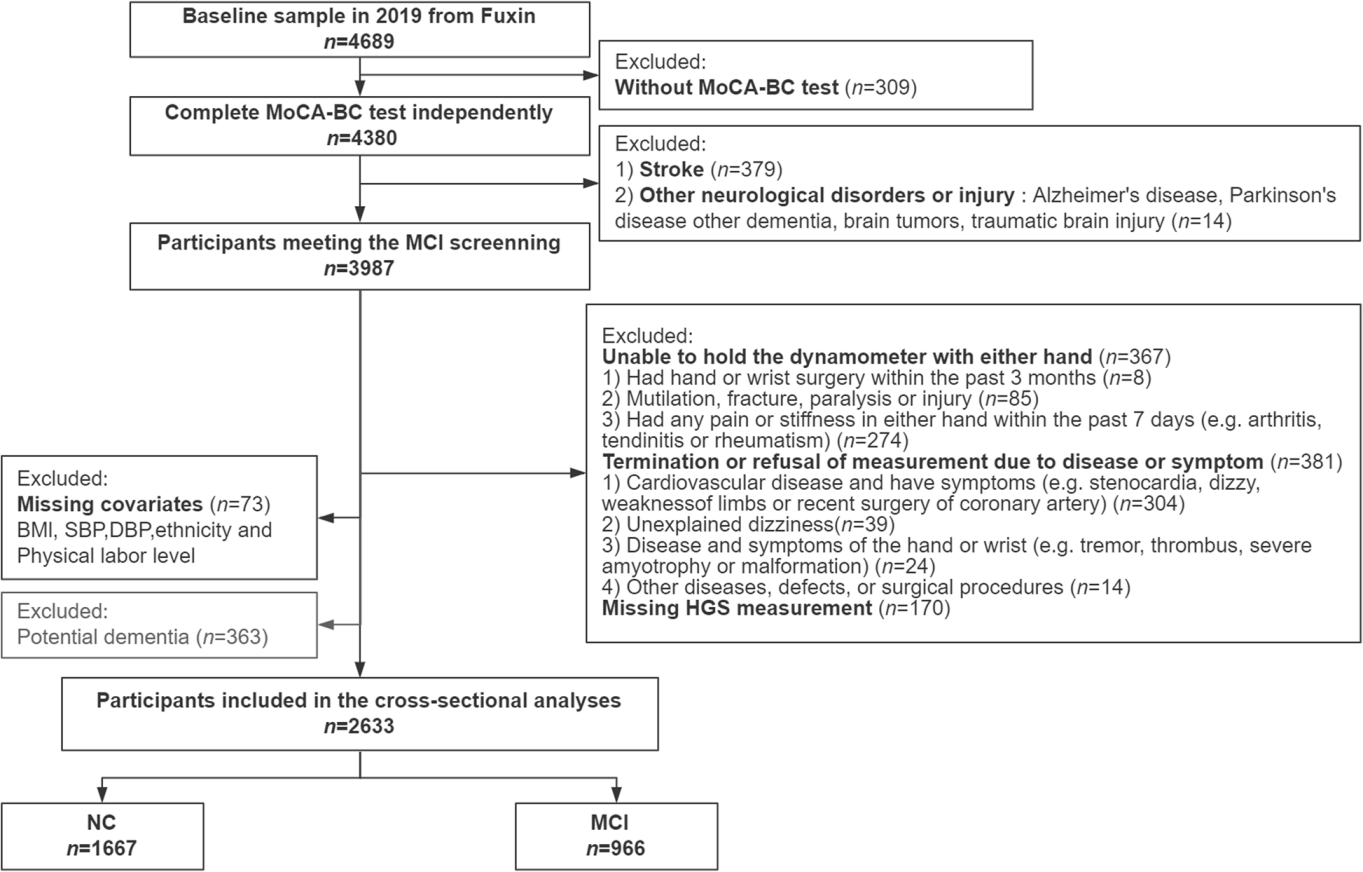
Flow diagram for the inclusion/exclusion of participants in the cross-sectional study. *MoCA-BC*, Chinese version of the Montreal Cognitive Assessment-Basic, *NC* Normal cognition, *MCI* Mild cognitive impairment

**Fig. 2:**
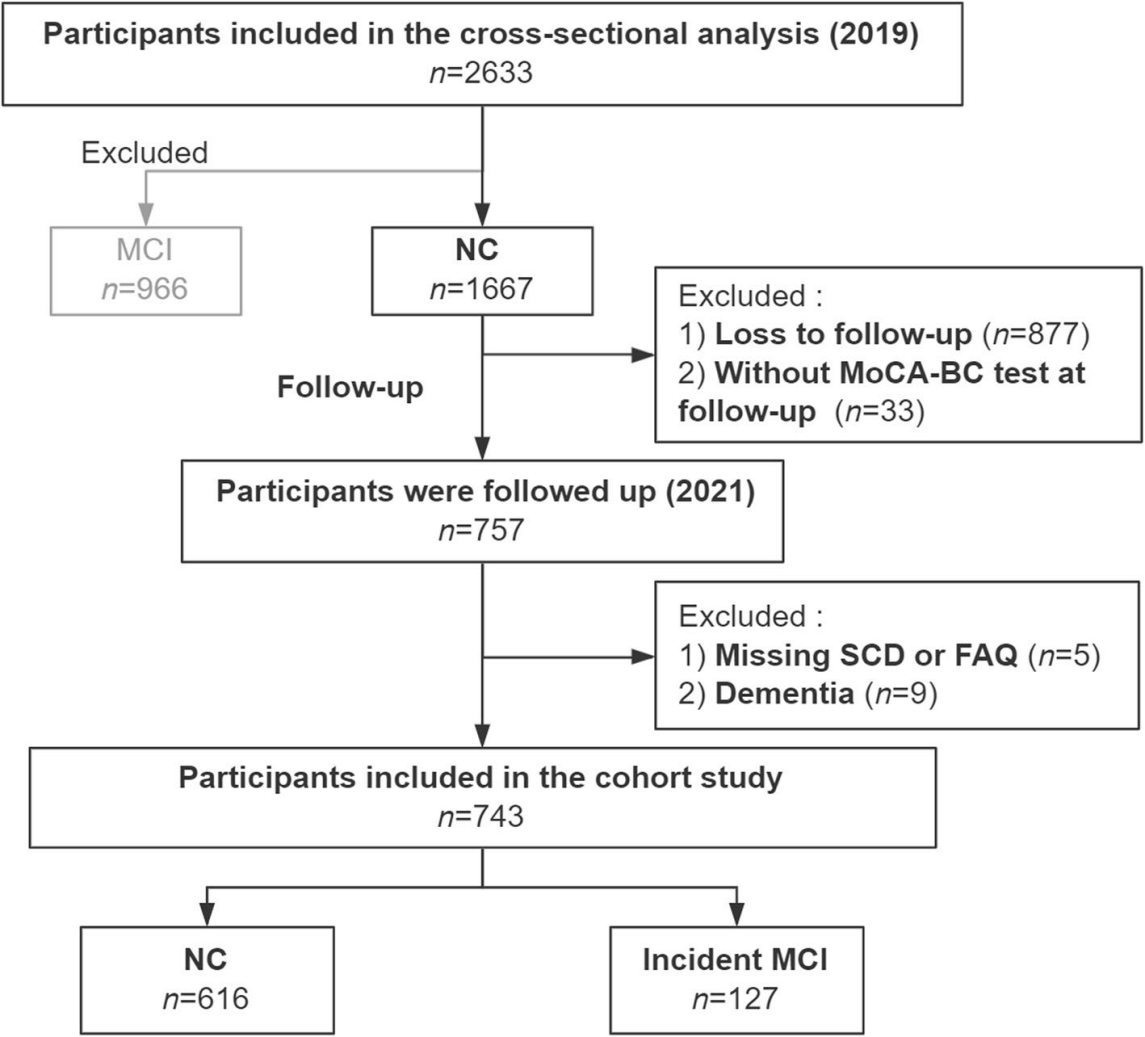
Flow diagram for the inclusion/exclusion of participants in longitudinal study. *MoCA-BC* Chinese version of the Montreal Cognitive Assessment-Basic, *NC* Normal cognition, *MCI* Mild cognitive impairment, *SCD* Subjective cognitive decline, *FAQ* Functional Activities Questionnaire

For the current analyses, those who independently completed the MoCA-BC test were included (Fig. 1). Then, we excluded the participants (1) with history of stroke and other mental disorders or brain diseases; (2) refusing or unable to participate in the grip strength measurement or missing the grip strength measurement (e.g., people with orthopedic disease or other severe disease, or missing data); (3) missing other covariates; (4) with potential dementia. Finally, 2633 participants were included in the cross-sectional analysis. Similarly, longitudinal data for incident MCI are shown in Fig. 2. The NCs at baseline who were followed up two years later and completed the cognitive assessment were included in the cohort study. Finally, 743 participants were enrolled in the longitudinal analysis.

### MoCA-BC test and diagnosis of MCI

The Chinese version of the Montreal Cognitive Assessment-Basic (MoCA-BC) is used to screen the MCI of Chinese elderly people with different education levels [19]. The scores were coded as NC, MCI and potential dementia stratified by years of education [19, 20]: (1) NC (19–30), MCI (13–18), and potential dementia (0–12) with ≤ 6 years of education; (2) NC (22–30), MCI (15–21), and potential dementia (0–14) with 7–12 years of education; (3) NC (24–30), MCI (16–23), and potential dementia (0–15) with > 12 years of education.

At baseline, we used MOCA-BC alone to screen for NC, MCI and potential dementia, and we excluded patients with potential dementia. In the process of follow-up, the diagnosis of MCI was based on criteria proposed by the National Institute on Aging-Alzheimer’s Association (NIA-AA) Working Group [21]. The criteria included the following: 1) cognitive complaints from subjects confirmed by an informant (relatives/doctors), which were assessed by the Subjective Cognitive Decline (SCD) scale; 2) objective evidence of impairment in one or more cognitive domains, which were assessed by MoCA-BC in this study; and 3) independence of daily functional ability, which was measured by the Functional Activities Questionnaire (FAQ). Scores ≥ 6 were suggested for dysfunction consistent with dementia divided from NC [22], and 4) not demented (based on the DSM-V) [23].

### Grip strength measurement

HGS was measured in kilograms (kg) using a dynamometer (Jamar Plus + , Patterson Medical, USA) and followed a standardized seated position protocol. The examiner instructed the participant to sit on a chair, place personal belongings aside, remove wrist jewelry, sit upright, and rest both feet naturally on the ground. One arm of the participant was positioned alongside the body, while the other hand grasped the dynamometer. The size of the dynamometer is adjusted based on the participant's hand size so that the participant can hold it with the second knuckle of the index finger at a 90° angle. During the test, the wrist and forearm should form a straight line, and the forearm should be at a 90° angle with the upper arm. Participants were encouraged to exert maximum force quickly and squeeze the dynamometer with maximum force for 3 s. Three measurements were taken for each hand, with a 30-s rest period between each measurement. The final result at the end of the testing session was recorded. In the current analysis, HGS was calculated using the average of both hands’ greatest force.

### Assessment of other variables

Data on demographic characteristics (age, sex, ethnicity, income, education, and marital status), lifestyle factors (smoking, drinking, and physical labor level) and history of disease (hypertension, diabetes, coronary heart disease, stroke, dementia, and depression) were collected by face-to-face interview with a standardized questionnaire. Body mass index was calculated as weight/height^2^ (kg/m^2^). The assessment of physical labor level was according to the National Standard of the People's Republic of China (GB3869-1997) [24], which was originally divided into four categories: (1) sitting posture: manual work or mild leg activity (under normal circumstances, such as typing, sewing, foot pedal operation, etc.); standing posture: operating equipment, controlling and inspecting devices, and primarily engaging in assembly work that requires upper arm strength; (2) continuous arm movements (such as sawing wood, etc.); work involving both arms and legs (such as operating trucks, tractors, or construction equipment); work involving both arms and torso (such as forging, operating pneumatic tools, painting, intermittent heavy lifting, weeding, hoeing, picking fruits and vegetables, etc.); (3) working involving both arms and torso exertion (such as lifting heavy objects, shoveling, hammering, sawing, planing, or chiseling hardwood, mowing, digging, etc.); (4) high-intensity digging and carrying, involving extremely intense activities performed at near maximum capacity. We combined 3 and 4 and reclassified them into three levels: "low", "moderate", and "high". According to WHO’s definition of smoking status in 1997 [25], smoking status in this study was defined as categorical variables (“Non-smoking”, “Former smoking” and “Current smoking”). Smokers were defined as those who had ever smoked at least one cigarette per day and continued for at least 6 months. Smokers were classified as “Former smoking” or “Current smoking” based on whether they had quit for more than 6 months. According to “the Physicians’ Guide to Helping Patients with Alcohol Problems,” which released by the National Institute on Alcohol Abuse and Alcoholism (NIAAA) in 1995 [26], alcohol intake in this study was defined as categorical variables (“Non-drinking”, “Former drinking” and “Current drinking”). Drinkers was defined as at least three drinks per week for 6 months. Then, drinkers were classified as “Former drinking” or “Current drinking” based on whether they had quit for more than 6 months. Diabetes mellitus was defined as fasting plasma glucose ≥ 7.0 mmol/L or the use of hypoglycemic drugs or insulin. Lipid abnormality was classified according to the Third Report of the National Cholesterol Education Program Expert Panel on Detection, Evaluation, and Treatment of High Blood Cholesterol in Adults final report [27]. According to the American Heart Association protocol [28], BP was measured three times at least 1 min after a rest of at least 5 min using a standardized automatic electronic blood pressure measuring instrument (HEM-8102A). For each participant, the BP was measured 3 times by trained research staff, and the average of three BP was used for the final analysis and evaluation. We defined hypertension as the use of antihypertensive medications in the last 2 weeks, DBP ≥ 90 mmHg or SBP ≥ 140 mmHg [29]. The definitions of depression, coronary heart disease and stroke were self-reported and confirmed by medical records.

### Statistical analysis

Continuous variables are presented as the mean ± standard deviation (SD), and categorical variables are expressed as percentages. Because of the asymmetric distribution, MoCA-BC is described by the median (interquartile range, IQR). ANOVA, Student’s t test, Mann–Whitney U test, and χ^2^ test are used to compare differences in continuous or categorical variables.

We considered HGS as a continuous variable and quintiles to enter the logistic regression models. We also conducted tests for trends between quintiles of HGS and the main outcomes based on variables containing median values for each quintile. As grip strength differed significantly between females and males, we ran fully adjusted models to test the main effects of HGS and an interaction between HGS and sex on MCI. The interaction term was significant between HGS and sex on incident MCI (Table S1), and all analyses were stratified by sex. Prior to multivariate analysis, we found no evidence of deviation from linearity between HGS and MCI using multivariate restricted cubic regression splines. We investigated the association between HGS and MCI using binary logistic regression models in the cross-sectional study. For the longitudinal data, we deleted the missing values and compared the baseline characteristics of those who were lost to follow-up with those who were followed up (Table S2-S3). We investigated the association between HGS and incident MCI using binary logistic regression models in the cohort study. To show the sex modification of the association between HGS and incident MCI, using the logistic regression model, we drew the fitting curve of HGS and ORs for incident MCI in males and females (Fig. 7).

For all analyses, the hierarchical modeling was used to add covariates in the following sequence: first, age was added, followed by ethnicity, education, marital status, income, body mass index, smoking, drinking, physical labor level, hypertension, diabetes, dyslipidemia and coronary heart disease. For the longitudinal analysis, since the baseline MOCA-BC score was a significant confounding factor, we included it in all models; due to the small sample size in each category of marital status (include 0) and the lack of statistical significance in the univariate analysis, we excluded marital status from the adjusted covariates. Subgroup analysis was also performed to divide the population into the elderly group (≥ 60 years) and the young and middle-aged group (35–60 years) in the cross-sectional analysis. In the cohort study, subgroup analysis was performed for people over 50 years, and we used the same diagnostic method of MCI consistent with the cross-sectional study (only the MoCA scale) and repeated the analysis as a sensitivity analysis. All statistical calculations were performed using R software (version 4.1.1). A 2-sided* P* value < 0.05 was considered statistically significant.

## Results

### Baseline characteristics

Baseline participants were on average 56.6 ± 9.8 years, 1713 (65.1%) were females, and the median (interquartile range, IQR) MoCA-BC was 22 (18, 26) points. The prevalence of MCI at baseline were 36.7%. The two-year cumulative incidence of MCI in the cohort study was 17.1%. As shown in Table 1, the average HGS was higher in males than in females (37.6 kg vs 24.4 kg, *P* < 0.001). Females had higher median MoCA-BC scores than males (22 [18, 25] vs 22 [19, 26], *P* = 0.007). There was a higher prevalence of MCI (41.0% vs. 34.3%) in males than it in females (*P* = 0.001). We also provided the baseline characteristics of NCs in the cohort study (Table S2-S3). After adjusting for confounding factors, the logistic regression model revealed that participants who were younger, male, of other ethnicities, engaged in low level of physical labor, and had lower baseline MoCA scores were more likely to be lost to follow-up in this study.

**Table 1:** Baseline sociodemographic characteristics stratified by sex

	**Total**	**Male**	**Female**	***P***
	*N* = 2633	*n* = 920	*n* = 1713	
**Age (y)**	56.6 ± 9.8	58.6 ± 9.6	55.6 ± 9.7	< 0.001^a^
**Age, ≥ 60y**	1074 (40.8)	448 (48.7)	626 (36.5)	< 0.001
**BMI (kg/m**^**2**^**)**	24.9 ± 3.7	24.5 ± 3.6	25.1 ± 3.8	< 0.001^a^
**Hypertension, yes**	994 (37.8)	400 (43.5)	594 (34.7)	< 0.001
**Diabetes, yes**	288 (10.9)	88 (9.6)	200 (11.7)	0.112
**Dyslipidemia, yes**	1220 (46.3)	424 (46.1)	796 (46.5)	0.884
**CHD, yes**	250 (9.5)	70 (7.6)	180 (10.5)	0.019
**Ethnicity**				0.277
Han	1672 (63.5)	597 (64.9)	1075 (62.8)	
Mongolian	846 (32.1)	290 (31.5)	556 (32.5)	
Others	115 (4.4)	33 (3.6)	82 (4.8)	
**Income**				0.047
< 10,000 yuan	1742 (66.2)	580 (63.0)	1162 (67.8)	
10,000–30000 yuan	713 (27.1)	272 (29.6)	441 (25.7)	
≥ 30,000 yuan	178 (6.8)	68 (7.4)	110 (6.4)	
**Education**				< 0.001
≤ Primary school	946 (35.9)	234 (25.4)	712 (41.6)	
Middle school	1250 (47.5)	479 (52.1)	771 (45.0)	
≥ High School	437 (16.6)	207 (22.5)	230 (13.4)	
**Marital status**				< 0.001
Married	2403 (91.3)	862 (93.7)	1541 (90.0)	
Widowhood	187 (7.1)	38 (4.1)	149 (8.7)	
Unmarried/Divorce	43 (1.6)	20 (2.2)	23 (1.3)	
**Smoking**				< 0.001
Non-smoking	1713 (65.1)	280 (30.4)	1433 (83.7)	
Current smoking	728 (27.6)	502 (54.6)	226 (13.2)	
Previous smoking	192 (7.3)	138 (15.0)	54 (3.2)	
**Drinking**				< 0.001
Non-drinking	1850 (70.3)	329 (35.8)	1521 (88.8)	
Current drinking	613 (23.3)	471 (51.2)	142 (8.3)	
Previous drinking	170 (6.5)	120 (13.0)	50 (2.9)	
**Physical labor level**				< 0.001
Low	723 (27.5)	200 (21.7)	523 (30.5)	
moderate	1786 (67.8)	635 (69.0)	1151 (67.2)	
High	124 (4.7)	85 (9.2)	39 (2.3)	
**HGS (kg)**	29.0 ± 9.0	37.6 ± 8.0	24.4 ± 5.4	< 0.001^a^
**MoCA-BC**^**c**^	22.0 [18.0, 26.0]	22.0 [18.0, 25.0]	22.0 [19.0, 26.0]	0.007 ^b^
**MCI, yes**	966 (36.7)	378 (41.1)	588 (34.3)	0.001

Data are median (interquartile range), mean (SD), or n (%); *P* value of categorical variables are calculated by *χ*^2^ test

^a^Student’s *t*-test

^b^Mann–Whitney *U* test

^c^MoCA-BC scores are presented as median (interquartile range)

*MoCA-BC* The Chinese version of Montreal Cognitive Assessment-Basic, *CHD* Coronary heart disease, *NC* Normal cognition, *MCI* Mild cognitive impairment, *HGS* Handgrip strength

### Prevalent MCI and incident MCI in different HGS groups

**Fig. 3:**
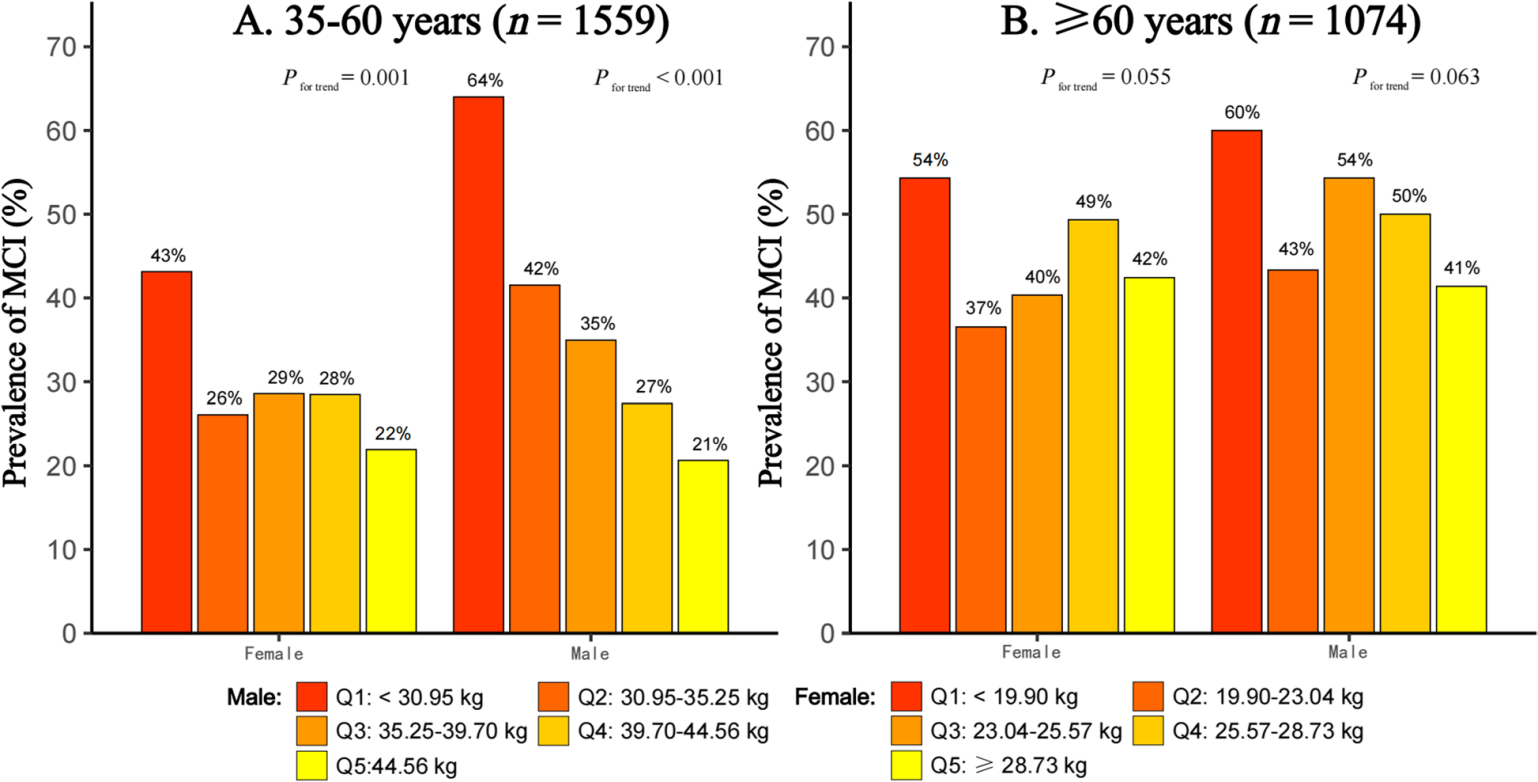
Prevalence of MCI in different quintiles of HGS stratified by **A**) aged < 60, **B**) aged ≥ 60. *MCI* Mild cognitive impairment, *HGS* Handgrip strength (HGS was divided into quintiles separately for males and females)

**Fig. 4:**
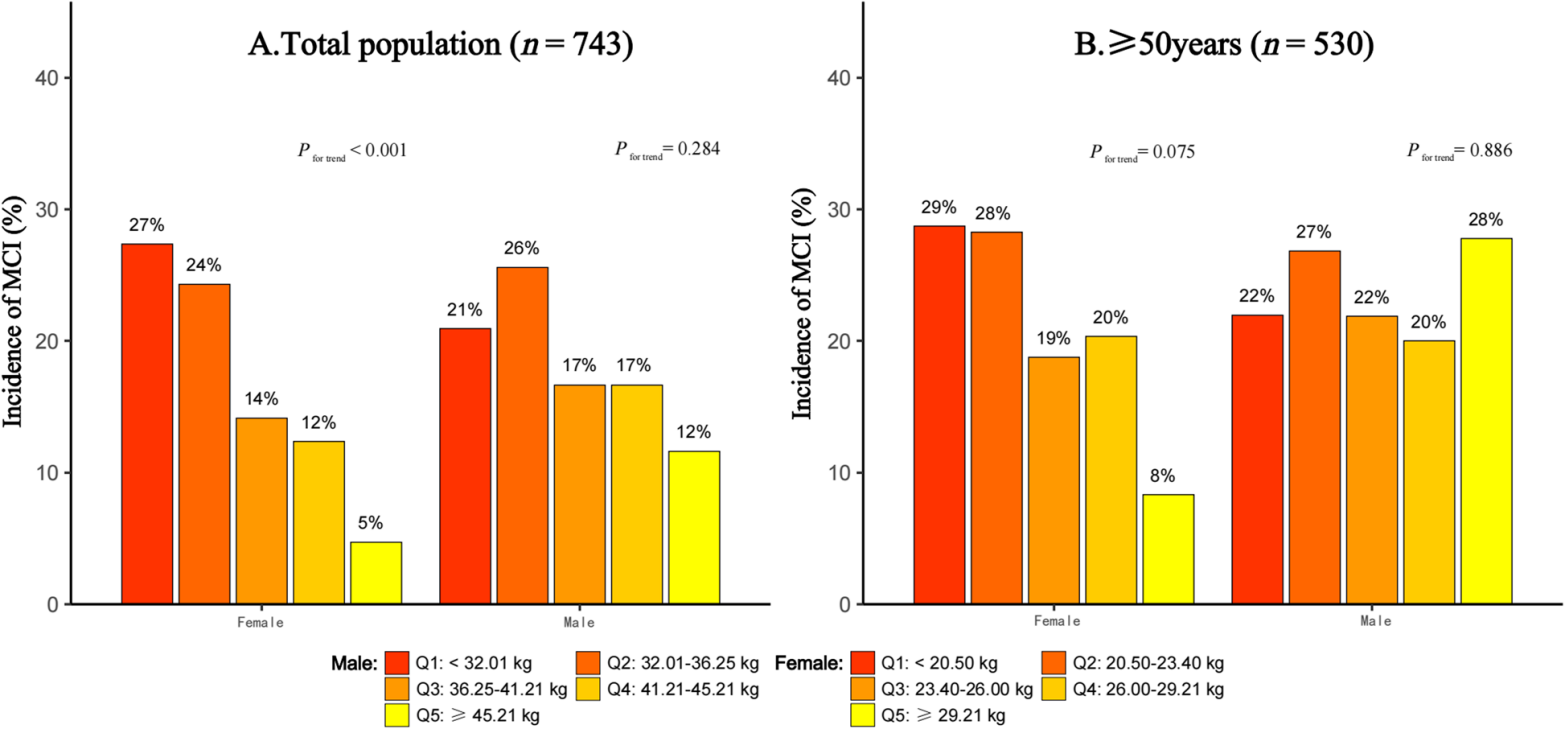
The two-year cumulative incidence of MCI stratified by sex and quintiles of HGS. *MCI* Mild cognitive impairment, HGS Handgrip strength (HGS was divided into quintiles separately for males and females)

Numbers of participants and min–max kilograms of each quintile in the cross-sectional study and the cohort study were shown in Table S4. The prevalence of MCI grouped by quintiles of HGS in males and females are shown in Fig. 3. The prevalence of MCI gradually decreased with increasing HGS level, and vice versa. The trend test showed statistical significance in the 35–60 age group (Female: *P*_for trend_ = 0.001, Male: *P*_for trend_ < 0.001), but not in the ≥ 60 age group (Female: *P*_for trend_ = 0.055, Male: *P*_for trend_ = 0.063). The cumulative incidence of MCI stratified by sex and quintiles of HGS was shown in Fig. 4. In the overall population (Fig. 4A), participants with lower HGS had a higher incidence of MCI in females (*P*_for trend_ < 0.001). However, in males, there was no statistically significant association between HGS level and the incidence of MCI (*P*_for trend_ = 0.284). In the population aged ≥ 50 years (Fig. 4B), although it did not reach statistical significance in females (*P*_for trend_ = 0.075), the incidence of MCI gradually decreased with increasing HGS level (incidence of MCI for quintiles of HGS was 29%, 28%, 19%, 20%, and 8%, respectively). In comparison, among males (*P*_for trend_ = 0.886), there was no significant association between HGS level and the incidence of MCI.

### Multivariate analysis of cross-sectional data

**Fig. 5:**
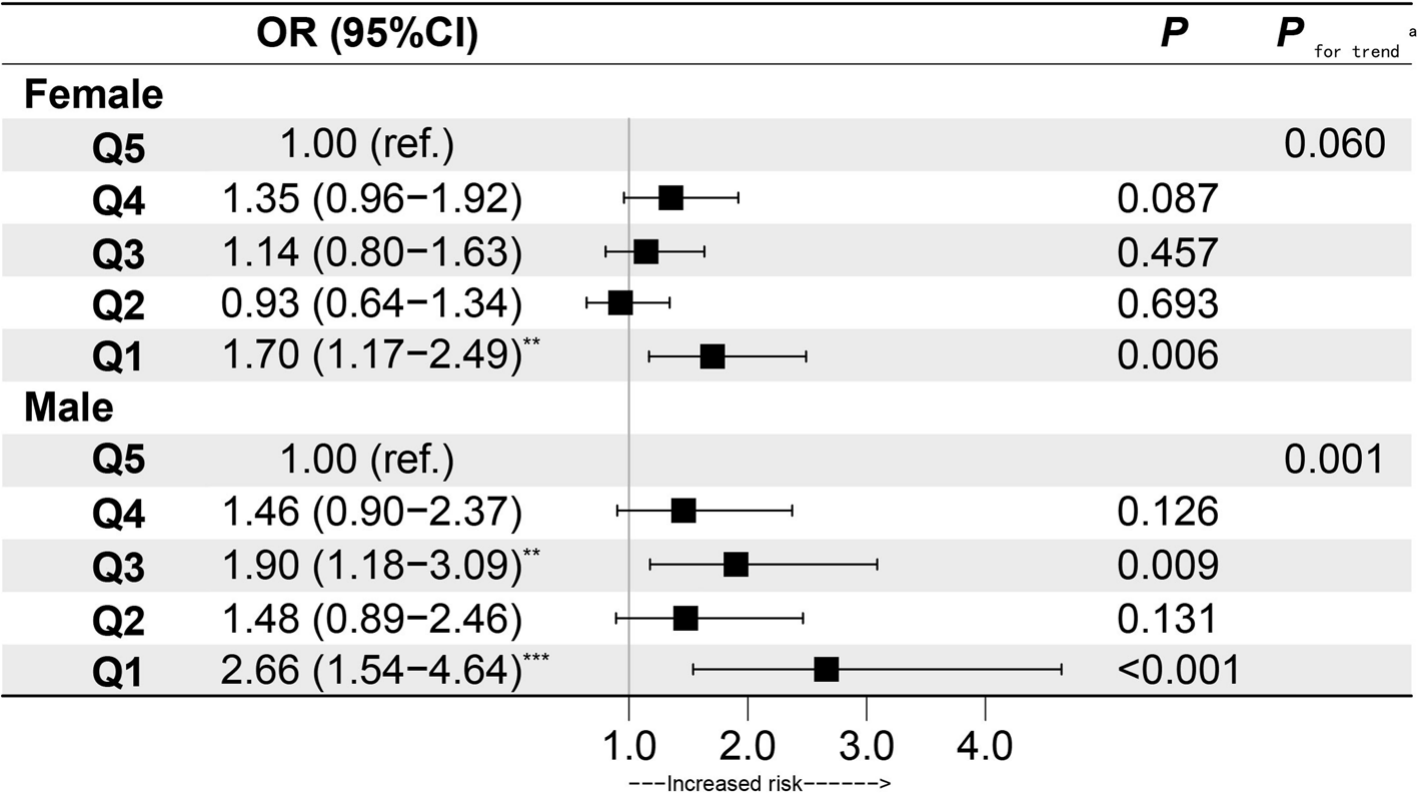
Adjusted ORs of baseline HGS levels for MCI in cross-sectional study. Adjusted: age, ethnicity, education, marital status, income, BMI, smoking, drinking, physical labor level, hypertension, diabetes, dyslipidemia and coronary heart disease. *OR* Odds ratio, *CI* Confidence interval, *MCI* Mild cognitive impairment, *HGS* Handgrip strength (HGS was divided to quintiles separately for males and females). a): Tests for trend are based on variables containing median values for each quintile

Table S5 presented the results of the hierarchical modeling, showing a decrease in the ORs after adjusting for age in both males and females. In the fully adjusted model, logistic regression models demonstrated that per 5 kg HGS decrease, there was an increased risk of incident MCI (OR: 1.19; 95% confidence interval [CI]: 1.06, 1.33) in males. No statistical significance was found between HGS and MCI (OR: 1.10; 95% CI: 0.98, 1.23) in females. In the fully adjusted model (Fig. 5), compared to the fifth quintile of HGS, the lowest HGS increased the risk of MCI (OR: 2.66; 95% CI: 1.54, 4.64) in males. In females, compared to the fifth quintile of HGS, the lowest HGS increased the risk of MCI (OR: 1.70; 95% CI: 1.17, 2.49). In Table S6, the total population was divided into the elderly group (≥ 60) and the young- and middle-aged group (35–60).

### Multivariate analysis of longitudinal data

**Fig. 6:**
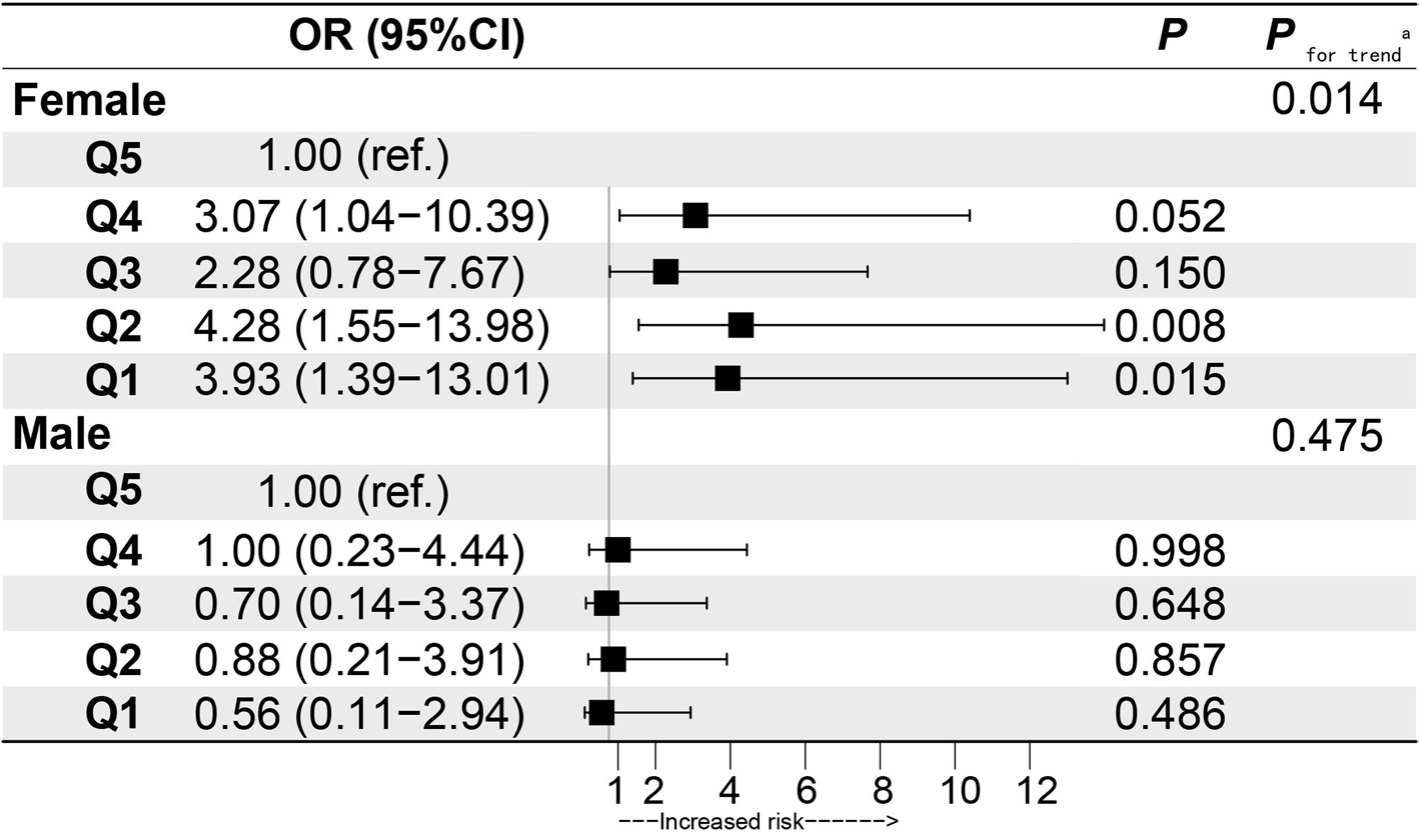
Adjusted ORs of baseline HGS levels for incident MCI in cohort study. Adjusted: age, baseline MOCA-BC score, ethnicity, education, income, BMI, smoking, drinking, physical labor level, hypertension, diabetes, dyslipidemia and coronary heart disease. *OR* Odds ratio, *MCI* Mild cognitive impairment, *HGS* Handgrip strength (HGS was divided into quintiles separately for males and females).a): Test for trend based on variables containing the median value for each quintile

**Fig. 7:**
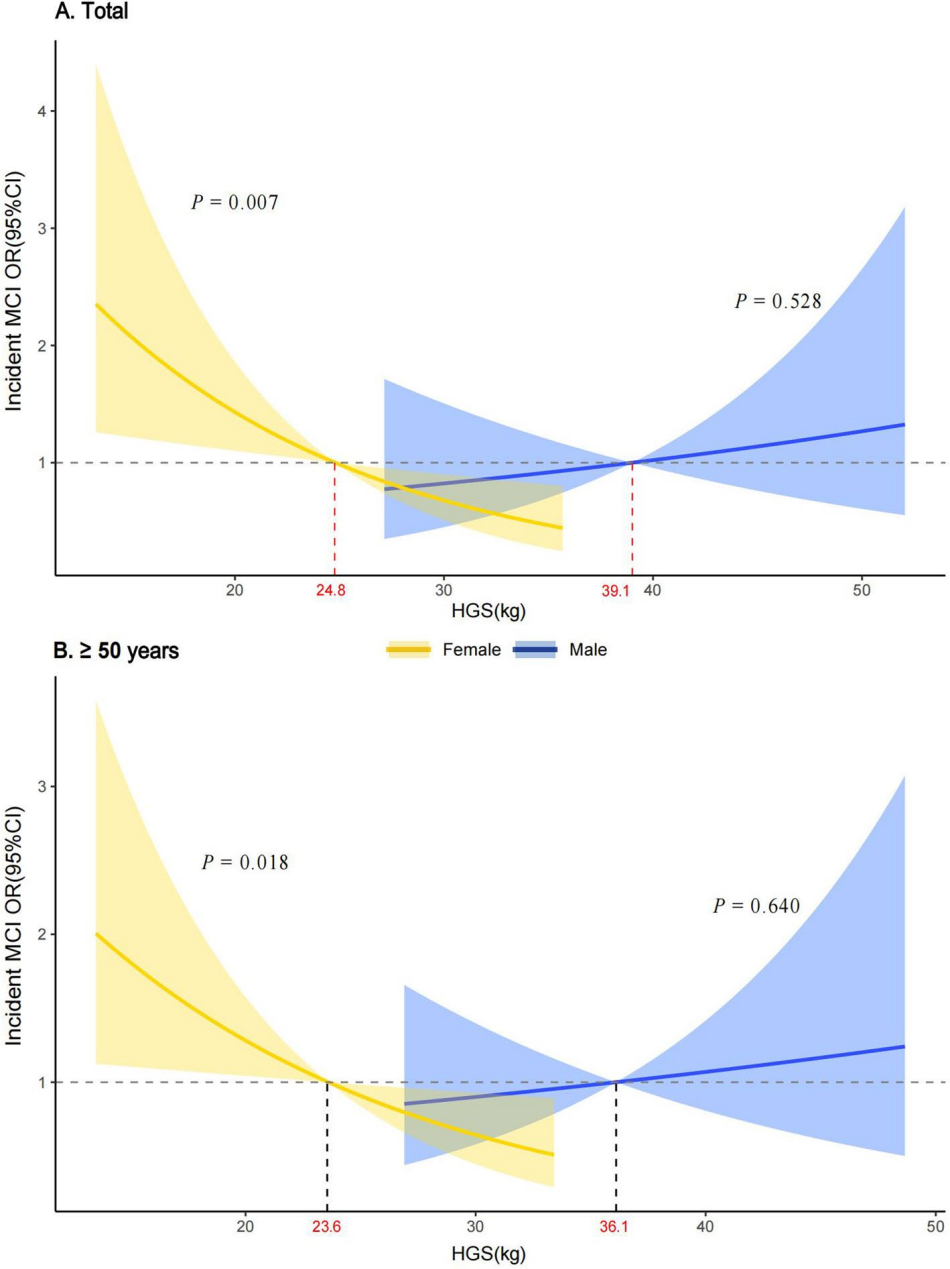
Sex difference in association of HGS with incident MCI in overall and ≥ 50 years group. The estimated OR of median HGS in male and female was set as the reference (OR = 1), and the logistic regression model was used to fit the change of ORs (95% CI) with the increase of HGS. The model was adjusted for age, baseline MOCA-BC score, ethnicity, education, income, BMI, smoking, drinking, physical labor level, hypertension, diabetes, dyslipidemia and coronary heart disease. *OR* Odds ratio, *CI* Confidence interval, *MCI* Mild cognitive impairment, *HGS* Handgrip strength

Adjusted for confounding variables, binary logistic regression models showed that no statistical significance was found between HGS and incident MCI (OR: 0.90; 95% CI: 0.64, 1.26) in males (Table S7). Simultaneously, no statistical significance was found between quintiles HGS and incident MCI (Fig. 6). However, per 5 kg HGS decrease, there was an increased risk of incident MCI in females (OR: 1.45; 95% CI: 1.11, 1.92). The lowest HGS increased the risk of incident MCI by approximately 4 times compared with the Fifth quintile of HGS (OR: 3.93; 95% CI: 1.39, 13.01). The analysis results remained consistent, when we used the same diagnostic method of MCI consistent with the cross-sectional study (Table S8-S9). Table S1 shows that the interaction term between sex and HGS (*P* = 0.015) on incident MCI was significant in the fully adjusted model. In total participants, Fig. 7A showed that ORs for incident MCI decreased significantly as HGS increased in females (*P* = 0.007) but remained flat in males (*P* = 0.528). In participants ≥ 50 years, similar findings were observed in Fig. 7B and in Table S10.

## Discussion

Several key findings emerged from this study. The study results demonstrated that 1) lower HGS is associated with a higher risk of MCI prevalence in this cross-sectional data. 2) In the cohort study, the association between HGS and incident MCI was modified by sex. NCs with lower HGS at baseline had an increased risk of incident MCI, however, this association was only found in females. These results remained consistent after adjusting for possible confounding factors.

Our cross-sectional study findings align with previous research [30–32]. A previous study from six low- and middle-income countries showed that, after adjusting for potential confounders, weak HGS was associated with an increased risk of MCI [30]. HGS was divided into quintiles to further verify the dose–response trend of this relationship in our study. A recent study suggested that cognitive changes occurred earlier than previously thought (before age 65) and were correlated with HGS [33]. However, this study was limited by its small sample size (51 participants) and convenience sampling. In our study, we included the general population (2633 participants ≥ 35 years), and we also found this association both in the older groups (≥ 60) and in the younger groups (35–60). We didn’t find cross-sectional studies that directly show the sex interaction effect between HGS and MCI.

Although the results of longitudinal studies are controversial, some previous longitudinal investigations have revealed that low HGS was associated with mild cognitive impairment in Americans [8, 12]. McGrath et al. [12] conducted a study analyzing 8 years of cohort data from the Health and Retirement Study (HRS) to investigate the association between HGS and the occurrence of cognitive impairments in 13,828 individuals aged 50 years and older in the United States. The study demonstrated that for every 5 kg decrease in HGS, there was an increased risk of mild cognitive impairment and severe cognitive impairment. A cohort study conducted in Chicago [8] also observed a correlation between HGS and an increased risk of AD and MCI. 1175 elderly individuals with an average age of 81 were recruited from over 40 geriatric institutions. The researchers assessed the presence of sarcopenia at baseline and followed up with the participants for an average of 5.6 years to evaluate the relationship between sarcopenia and the incidence of MCI and AD. The researchers performed separate analyses for the three indicators of sarcopenia as well as various combinations. The study revealed that even after adjusting for muscle mass, grip strength remained associated with the occurrence of MCI and AD, while muscle mass consistently showed no association with MCI and AD. This suggests that the decline in muscle strength, rather than muscle mass, serves as a predictive factor for the onset of MCI and dementia. Another cohort study conducted in Korea[15] showed that among 297 elderly individuals aged 65 years and above, slow walking speed was associated with an increased risk of MCI (HR = 2.22; 95% CI: 1.05, 4.72, *P* = 0.038). Furthermore, weak grip strength was found to increase the risk of MCI by 34%, although this increase was not statistically significant (HR = 1.337; 95% CI: 0.28, 6.37, *P *= 0.716). Clearly, our cohort results support the idea that grip strength may be a predictor of MCI in rural areas of China, at least in females.

Interestingly, our longitudinal results showed a stronger association between HGS and incident MCI in females than in males, whereas the opposite pattern was observed in the cross-sectional study. Similarly, McGrath et al. [12] revealed that a significant correlation between 5 kg decrease in grip strength and increased risk of severe cognitive impairment in women (OR = 1.25; 95% CI: 1.05, 1.49), while no statistical significance was observed in men (OR = 1.06; 95% CI: 0.90, 1.26). We believe that this is mainly due to the higher "cognitive reserve" of female compared to male, resulting in more NCs in females with neurostructural injury. Most studies suggest that association between grip strength and MCI may be explained by common age-related factors [11] (oxidative stress, inflammation, hormonal levels and so on), which affect both muscle and the brain. The association between grip strength and MCI may also be attributed to physical exercise. Regular physical exercise leads to higher muscle strength, and exercise has been shown to improve cognitive function [34, 35]. Mechanistically, the neurovascular adaptations induced by exercise in older adults promote cognitive enhancement through stimulation of neurogenesis, angiogenesis, synaptic plasticity, and reduction of pro-inflammatory processes and cellular damage caused by oxidative stress [36]. Furthermore, the "massive redeployment" hypothesis [37, 38] proposes that the process of human cognitive evolution is akin to component reuse in software engineering, where the evolution of higher-level cognition occurs not through the generation of new structural regions but through the reallocation and reassembly of relevant "lower-level structural regions." The motor neural system is linked to the prefrontal and parietal regions and their interconnections [39], implying that the control of muscle strength and coordination in these regions may be closely associated with the formation of various higher-level cognitive functions. Grip strength decline may be an early manifestation of decreased physical activity and coordination-related neural regions, followed by subsequent impacts on "higher-level cognitive domains" as the damage progresses. In summary, one plausible explanation for the association between grip strength and MCI is the compromised neural-muscular relationship at the structural level. However, impairment in cognitive performance does not always align with neural damage. "Cognitive reserve" [40] describes the capacity of the brain's effectiveness and plasticity to buffer the disabling effects of neuropathology, allowing individuals to cope successfully with brain pathology. Studies have shown that individuals with higher cognitive reserve, even in the presence of cognitive impairment pathology and neuroimaging evidence, do not exhibit cognitive deficits [16, 41]. Female naturally possess higher cognitive reserve than male [42, 43], which influences the duration of preclinical stage before cognitive changes. During this period, the brain is already exposed to risk factors associated with cognitive impairment and dementia, which also affect the neural structures involved in movement and physical coordination or directly damage the muscles. In the cross-sectional study, there will be a higher proportion of females who are protected by cognitive reserve and do not develop MCI. However, their neural structures may still be damaged to varying degrees and may be associated with poorer grip strength. As a result, the association between grip strength and MCI is weaker in females compared to males. In the longitudinal study, among cognitively normal individuals, baseline grip strength can better predict the occurrence of MCI in females compared to males, due to the higher proportion of neural damage in females. However, no studies have directly demonstrated this association, and pathological or radiological examinations should be performed to further confirm this phenomenon.

Our study revealed that females with low HGS have a more than threefold increased risk of developing MCI within two years in rural China. Female's higher cognitive reserve may potentially bring them more risk in the future. Researches [17, 44] suggested that female experienced faster cognitive decline after developing cognitive impairment or dementia compared to men, and it is particularly important to combine the early risk indicators of cognitive impairment, such as HGS, as imaging information is not available in those backward areas. The grip strength measurement is simple, quick and very cheap. However, the sex difference in development of MCI still require further confirmation, especially considering that our study represents a rural population in northeastern China with lower average education levels and income. Future researches need to replicate our findings in broader populations, including urban and other regions. Additionally, incorporating potential pathological or imaging data and conducting animal experiments would further confirm it.

The advantages of our research are as follows: 1) our study was based on a large sample of the general population, and evidence was strengthened by the cross-sectional and longitudinal cohort study; 2) MoCA-BC is more suitable for the Chinese rural population with a low education level; and 3) there is no longitudinal study that provides evidence of a relationship between HGS and cognition in rural China. However, there are several noteworthy limitations to this study. First, our study did not examine the dynamic changes in grip strength that occurred earlier, and future research is needed to explore this association. Second, we did not have imaging information to further confirm whether the sex difference in this association was due to cognitive reserve. However, whether confirmed by imaging information or not, our results can still provide clinical and public health implications. Third, the definition of MCI at baseline were only using the MoCA-BC, which reduced the specificity for the diagnosis of MCI. This may underestimate the results in our study. Nonetheless, NCs initially included in the cohort can be guaranteed. Finally, our findings are subject to the limitations of an observational study design. Due to the low follow-up rate, it may cause the follow-up bias in the study. However, our drop-out analyses showed that the demographic characteristics of participants and non-participants in follow-up were highly similar, except for age and sex. Our results can be considered relatively reliable after adjusting for confounding factors. Nevertheless, more studies are expected to validate the results of this study in the future.

## Conclusions

Grip strength is associated with MCI. And this study suggests that incident MCI can be predicted and detected by grip strength earlier in females. This study could provide some guidance in rural and poor areas to alert women who have lower grip strength, even though they don't seem to have any cognitive problems, they may be experiencing cognitive impairment. Future researches are needed to replicate our findings in broader populations and should focus on understanding the biological mechanism of this relationship between HGS and MCI.

### Supplementary Information

**Additional file 1. **

## Data Availability

The data that support the findings of this study are restricted, which were used under license for the current study and thus are not publicly available.

## Abbreviations

AD: Alzheimer's disease
CI: Confidence interval
DALYs: Disability adjusted life years
FAQ: Functional Activities Questionnaire
HGS: Hand grip strength
IQR: Interquartile range
MCI: Mild cognitive impairment
MOCA-BC: The Chinese version of Montreal Cognitive Assessment-Basic
NCs: Cognitively normal individuals
NIA-AA: National Institute on Aging-Alzheimer’s Association
OR: Odds ratio
SCD: Subjective Cognitive Decline
SD: Standard deviation

## References

[1] Organization WH. Global status report on the public health response to dementia. https://www.who.int/publications/i/item/9789240033245. Accessed 1 Sept 2021.

[2] Sanz-Blasco R, Ruiz-Sánchez de León JM, Ávila-Villanueva M, Valentí-Soler M, Gómez-Ramírez J, Fernández-Blázquez MA (2021). Transition from mild cognitive impairment to normal cognition: Determining the predictors of reversion with multi-state Markov models. Alzheimer's Dementia.

[3] (2016) 2016 Alzheimer's disease facts and figures. Alzheimer's & dementia : the journal of the Alzheimer's Association 12(4):459–509. 10.1016/j.jalz.2016.03.001. 10.1016/j.jalz.2016.03.001 27570871

[4] Lim U, Wang S, Park SY (2021). Risk of Alzheimer's disease and related dementia by sex and race/ethnicity: The Multiethnic Cohort Study. Alzheimer's Dementia.

[5] Oveisgharan S, Arvanitakis Z, Yu L, Farfel J, Schneider JA, Bennett DA (2018). Sex differences in Alzheimer's disease and common neuropathologies of aging. Acta Neuropathol.

[6] Bullain SS, Corrada MM, Shah BA, Mozaffar FH, Panzenboeck M, Kawas CH (2013). Poor physical performance and dementia in the oldest old: the 90+ study. JAMA Neurol.

[7] Sabia S, Dugravot A, Dartigues JF (2017). Physical activity, cognitive decline, and risk of dementia: 28 year follow-up of Whitehall II cohort study. BMJ (Clinical research ed).

[8] Beeri MS, Leugrans SE, Delbono O, Bennett DA, Buchman AS (2021). Sarcopenia is associated with incident Alzheimer's dementia, mild cognitive impairment, and cognitive decline. J Am Geriatr Soc.

[9] Kikkert LHJ, Vuillerme N, van Campen JP, Hortobágyi T, Lamoth CJ (2016). Walking ability to predict future cognitive decline in old adults: A scoping review. Ageing Res Rev.

[10] Bodilsen AC, Juul-Larsen HG, Petersen J, Beyer N, Andersen O, Bandholm T (2015). Feasibility and inter-rater reliability of physical performance measures in acutely admitted older medical patients. PLoS ONE.

[11] Fritz NE, McCarthy CJ, Adamo DE (2017). Handgrip strength as a means of monitoring progression of cognitive decline - A scoping review. Ageing Res Rev.

[12] McGrath R, Robinson-Lane SG, Cook S (2019). Handgrip Strength Is Associated with Poorer Cognitive Functioning in Aging Americans. Journal of Alzheimer's disease : JAD.

[13] Albala C, Lera L, Sanchez H (2017). Frequency of frailty and its association with cognitive status and survival in older Chileans. Clin Interv Aging.

[14] Salinas-Rodríguez A, Palazuelos-González R, Rivera-Almaraz A, Manrique-Espinoza B (2021). Longitudinal association of sarcopenia and mild cognitive impairment among older Mexican adults. J Cachexia Sarcopenia Muscle.

[15] Moon JH, Moon JH, Kim KM (2016). Sarcopenia as a Predictor of Future Cognitive Impairment in Older Adults. J Nutr Health Aging.

[16] Sundermann EE, Biegon A, Rubin LH (2016). Better verbal memory in women than men in MCI despite similar levels of hippocampal atrophy. Neurology.

[17] Duarte-Guterman P, Albert AY, Barha CK, Galea LAM, On Behalf Of The Alzheimer's Disease Neuroimaging I (2021). Sex influences the effects of APOE genotype and Alzheimer's diagnosis on neuropathology and memory. Psychoneuroendocrinology.

[18] Rothwell ES, Workman KP, Wang D, Lacreuse A (2022). Sex differences in cognitive aging: a 4-year longitudinal study in marmosets. Neurobiol Aging.

[19] Chen KL, Xu Y, Chu AQ (2016). Validation of the Chinese Version of Montreal Cognitive Assessment Basic for Screening Mild Cognitive Impairment. J Am Geriatr Soc.

[20] Huang L, Chen KL, Lin BY (2018). Chinese version of Montreal Cognitive Assessment Basic for discrimination among different severities of Alzheimer's disease. Neuropsychiatr Dis Treat.

[21] Albert MS, DeKosky ST, Dickson D (2011). The diagnosis of mild cognitive impairment due to Alzheimer's disease: recommendations from the National Institute on Aging-Alzheimer's Association workgroups on diagnostic guidelines for Alzheimer's disease. Alzheimer's Dementia.

[22] Yin L, Ren Y, Wang X (2020). The power of the Functional Activities Questionnaire for screening dementia in rural-dwelling older adults at high-risk of cognitive impairment. Psychogeriatrics : the official journal of the Japanese Psychogeriatric Society.

[23] American Psychiatric Association D-TF. Diagnostic and statistical manual of mental disorders: DSM-5™ (5th ed.). American Psychiatric Publishing. 10.1176/appi.books.9780890425596.

[24] China State Bureau of Technical Supervision (1997). Classification on intensity of physical work: GB 3869–1997. https://openstd.samr.gov.cn/bzgk/gb/index.

[25] Organization WH. Guidelines for controlling and monitoring the tobacco epidemic. 1998. https://apps.who.int/iris/handle/10665/42049.

[26] NIAAA. The Physicians' Guide to Helping Patients With Alcohol Problems. NIH Publication; 1995:3769.

[27] (2001) Executive Summary of The Third Report of The National Cholesterol Education Program (NCEP) Expert Panel on Detection, Evaluation, And Treatment of High Blood Cholesterol In Adults (Adult Treatment Panel III). Jama 285(19):2486–97. 10.1001/jama.285.19.2486. 10.1001/jama.285.19.2486 11368702

[28] O'Brien E, Petrie J, Littler W (1990). The British Hypertension Society protocol for the evaluation of automated and semi-automated blood pressure measuring devices with special reference to ambulatory systems. J Hypertens.

[29] (2019) 2018 Chinese Guidelines for Prevention and Treatment of Hypertension-A report of the Revision Committee of Chinese Guidelines for Prevention and Treatment of Hypertension. Journal of geriatric cardiology : JGC 16(3):182–241. 10.11909/j.issn.1671-5411.2019.03.014. 10.11909/j.issn.1671-5411.2019.03.014 PMC6500570 31080465

[30] Vancampfort D, Stubbs B, Firth J, Smith L, Swinnen N, Koyanagi A (2019). Associations between handgrip strength and mild cognitive impairment in middle-aged and older adults in six low- and middle-income countries. Int J Geriatr Psychiatry.

[31] Cui M, Zhang S, Liu Y, Gang X, Wang G (2021). Grip Strength and the Risk of Cognitive Decline and Dementia: A Systematic Review and Meta-Analysis of Longitudinal Cohort Studies. Frontiers in aging neuroscience.

[32] Pérez-Sousa M, Del Pozo-Cruz J, Olivares PR, Cano-Gutiérrez CA, Izquierdo M, Ramírez-Vélez R (2021) Role for Physical Fitness in the Association between Age and Cognitive Function in Older Adults: A Mediation Analysis of the SABE Colombia Study. International journal of environmental research and public health 18(2)10.3390/ijerph18020751. 10.3390/ijerph18020751 PMC7829928 33477293

[33] Adamo DE, Anderson T, Koochaki M, Fritz NE (2020). Declines in grip strength may indicate early changes in cognition in healthy middle-aged adults. PLoS ONE.

[34] Prakash RS, Voss MW, Erickson KI, Kramer AF (2015). Physical activity and cognitive vitality. Annu Rev Psychol.

[35] Angevaren M, Aufdemkampe G, Verhaar HJ, Aleman A, Vanhees L (2008) Physical activity and enhanced fitness to improve cognitive function in older people without known cognitive impairment. The Cochrane database of systematic reviews (3):Cd005381. 10.1002/14651858.CD005381.pub3. 10.1002/14651858.CD005381.pub3 18646126

[36] Rasmussen P, Brassard P, Adser H (2009). Evidence for a release of brain-derived neurotrophic factor from the brain during exercise. Exp Physiol.

[37] Anderson M (2007). The massive redeployment hypothesis and the functional topography of the brain. Brain..

[38] Anderson ML (2007). Evolution of cognitive function via redeployment of brain areas. The Neuroscientist : a review journal bringing neurobiology, neurology and psychiatry.

[39] Heuninckx S, Wenderoth N, Debaere F, Peeters R, Swinnen SP (2005). Neural basis of aging: the penetration of cognition into action control. The Journal of neuroscience : the official journal of the Society for Neuroscience.

[40] Stern Y (2012). Cognitive reserve in ageing and Alzheimer's disease. The Lancet Neurology.

[41] Stern Y, Gurland B, Tatemichi TK, Tang MX, Wilder D, Mayeux R (1994). Influence of education and occupation on the incidence of Alzheimer's disease. JAMA.

[42] Sundermann EE, Maki PM, Rubin LH, Lipton RB, Landau S, Biegon A (2016). Female advantage in verbal memory: Evidence of sex-specific cognitive reserve. Neurology.

[43] Illán-Gala I, Casaletto KB, Borrego-Écija S (2021). Sex differences in the behavioral variant of frontotemporal dementia: A new window to executive and behavioral reserve. Alzheimer's Dementia.

[44] Hall CB, Derby C, LeValley A, Katz MJ, Verghese J, Lipton RB (2007). Education delays accelerated decline on a memory test in persons who develop dementia. Neurology.

