# Supplementary material for: Sex-modified association between grip strength and mild cognitive impairment: a cross-sectional and follow-up study in rural China
Source: BMC Geriatr. 2023 Nov 2;23:710. doi: 10.1186/s12877-023-04376-1 (PMC10623700; doi:10.1186/s12877-023-04376-1)
Supplement: Supplementary file 1 — Additional file 1. [file 12877_2023_4376_MOESM1_ESM.docx]

| **Table S1. Interaction between HGS and sex on the incident MCI in cohort study** | | | |
| --- | --- | --- | --- |
| **Model/variable** | | **OR (95% CI)** | ***P*** |
| **Main effect model ^a^** | |  |  |
|  | **HGS (per 5kg decrease)** | 1.17 (0.95-1.43) | 0.133 |
|  | **Sex (male)** | 0.89 (0.40-1.98) | 0.784 |
| **Interaction term included ^a^** | |  |  |
|  | **HGS (per 5kg decrease)** | 1.42 (1.10-1.86) | **0.008** |
|  | **Sex (male)** | 0.06 (0.01-0.62) | **0.018** |
|  | **HGS×sex** | 0.65 (0.45-0.92) | **0.015** |

**a** The models were adjusted for age, baseline MOCA-BC score, ethnicity, education, income, BMI, smoking, drinking, physical labor level, hypertension, diabetes, dyslipidemia and coronary heart disease; OR, odds ratio; CI, confidence interval; MCI, mild cognitive impairment; HGS, handgrip strength (HGS was divided to quintile separately for males and females)

| **Table S2. socio-demographic characteristics of NCs as well as those loss to follow up in cohort study** | | | | | | | |
| --- | --- | --- | --- | --- | --- | --- | --- |
| **Variable** | | Baseline NCs |  | Followed up |  | Lost to follow up | P |
|  |  | *n*=1667 |  | n=752 |  | n=915 |  |
| **Age (years)** | | 54.8±9.8 |  | 56.0±9.6 |  | 53.7±9.8 | **<0.001** a |
| **BMI (kg/m^2^)** | | 25.01±3.66 |  | 25.13±3.67 |  | 24.91±3.65 | 0.222 a |
| **Sex, male** | | 542 (32.5) |  | 217 (28.9) |  | 325 (35.5) | **0.005** |
| **Hypertension, yes** | | 591 (35.5) |  | 279 (37.1) |  | 312 (34.1) | 0.221 |
| **Diabetes, yes** | | 167 (10.0) |  | 90 (12.0) |  | 77 (8.4) | **0.020** |
| **Dyslipidemia, yes** | | 776 (46.6) |  | 350 (46.5) |  | 426 (46.6) | 1.00 |
| **CHD, yes** | | 137 (8.2) |  | 70 (9.3) |  | 67 (7.3) | 0.168 |
| **Ethnicity** | |  |  |  |  |  | 0.087 |
|  | Han | 1054 (63.2) |  | 475 (63.2) |  | 579 (63.3) |  |
|  | Mongolian | 528 (31.7) |  | 248 (33.0) |  | 280 (30.6) |  |
|  | Others | 85 (5.1) |  | 29 (3.9) |  | 56 (6.1) |  |
| **Income** | |  |  |  |  |  | **0.017** |
|  | <10000 yuan | 1074 (64.4) |  | 511 (68.0) |  | 563 (61.5) |  |
|  | 10000-30000 yuan | 472 (28.3) |  | 196 (26.1) |  | 276 (30.2) |  |
|  | ≥30000 yuan | 121 (7.3) |  | 45 (6.0) |  | 76 (8.3) |  |
| **Education** | |  |  |  |  |  | 0.492 |
|  | ≤ Primary school | 525 (31.5) |  | 247 (32.8) |  | 278 (30.4) |  |
|  | Middle school | 834 (50.0) |  | 365 (48.5) |  | 469 (51.3) |  |
|  | ≥ High School | 308 (18.5) |  | 140 (18.6) |  | 168 (18.4) |  |
| **Marital status** | |  |  |  |  |  | 0.771 |
|  | Married | 1540 (92.4) |  | 691 (91.9) |  | 849 (92.8) |  |
|  | Widowhood | 101 (6.1) |  | 49 (6.5) |  | 52 (5.7) |  |
|  | Unmarried/Divorce | 26 (1.6) |  | 12 (1.6) |  | 14 (1.5) |  |
| **Smoking** | |  |  |  |  |  | 0.058 |
|  | Non-smoking | 1134 (68.0) |  | 529 (70.3) |  | 605 (66.1) |  |
|  | Current smoking | 430 (25.8) |  | 173 (23.0) |  | 257 (28.1) |  |
|  | Previous smoking | 103 (6.2) |  | 50 (6.6) |  | 53 (5.8) |  |
| **Drinking** | |  |  |  |  |  | 0.146 |
|  | Non-drinking | 1193 (71.6) |  | 556 (73.9) |  | 637 (69.6) |  |
|  | Current drinking | 372 (22.3) |  | 155 (20.6) |  | 217 (23.7) |  |
|  | Previous drinking | 102 (6.1) |  | 41 (5.5) |  | 61 (6.7) |  |
| **Physical labor level** | |  |  |  |  |  | 0.156 |
|  | Low | 444 (26.6) |  | 184 (24.5) |  | 260 (28.4) |  |
|  | moderate | 1126 (67.5) |  | 526 (69.9) |  | 600 (65.6) |  |
|  | High | 97 (5.8) |  | 42 (5.6) |  | 55 (6.0) |  |
| HGS (kg) | | 29.61±9.14 |  | 28.86±8.93 |  | 30.23±9.27 | **0.002** a |
| **Baseline MoCA-BC**c | | 25.00 [22.00, 27.00] |  | 25.00 [22.00, 27.00] |  | 25.00 [22.00, 27.00] | 0.57 b |
| Data are median (InterQuartile Range), mean±SD, or n (%); P-value of categorical variables are calculated by χ2 test; a): Student’s t-test b): Mann–Whitney U test; c): MoCA-BC scores are presented as median (interquartile range) MoCA-BC, Montreal Cognitive Assessment-Basic for Chinese; NCs, cognitively normal individuals; CHD, coronary heart disease; HGS, hand grip strength. | | | | | | | |

| **Table S3. Baseline characteristics of the lost population using multivariate logistic model in cohort study ^a^** | | | | |
| --- | --- | --- | --- | --- |
| **Variable** | | | **OR(95%CI)** | ***P*** |
| **Age (y)** | | | 0.97 (0.95-0.98) | **<0.001** |
| **Sex, male** | | | 1.62 (1.08-2.44) | **0.019** |
| **Ethnicity** | | |  |  |
|  | Han | | 1.00 (Ref.) |  |
|  | Mongolian | | 1.02 (0.82-1.27) | 0.821 |
|  | Others | | 1.79 (1.11-2.97) | **0.020** |
| Physical labor level | | |  |  |
|  | | Low | 1.00 (Ref.) |  |
|  | | moderate | 0.74 (0.58-0.94) | **0.012** |
|  | | High | 0.76 (0.48-1.23) | 0.269 |
| **Baseline MoCA-BC** | | | 0.96 (0.92-0.99) | **0.029** |

**a** Whether lost the follow-up was used as the dependent variable, and the independent variables included HGS, age, sex, ethnicity, education, marital status, income, BMI, smoking, drinking, physical labor level, hypertension, diabetes, dyslipidemia, coronary heart disease and baseline MoCA-BC. The results only showed statistically significant variables.

MoCA-BC, Montreal Cognitive Assessment-Basic for Chinese; OR, odds ratio; CI, confidence interval; Ref., reference

| **Table S4. Numbers of participants and min-max kilograms of each quintile in males and females in cross-sectional study and cohort study** | | | | | |
| --- | --- | --- | --- | --- | --- |
| **HGS** | **Male** | |  | **Female** | |
|  | **Range of HGS** | ***n* (%)** |  | **Range of HGS** | ***n* (%)** |
| **Cross-sectional** **study** |  |  |  |  |  |
| Q1 | < 30.95 kg | 185 (20.1%) |  | < 19.90 kg | 345 (20.1%) |
| Q2 | 30.95 kg-35.25 kg | 185 (20.1%) |  | 19.90 kg-23.04 kg | 340 (19.8%) |
| Q3 | 35.25 kg-39.70 kg | 184 (20.0%) |  | 23.04 kg-25.57 kg | 343 (20.0%) |
| Q4 | 39.70 kg-44.56 kg | 182 (19.8%) |  | 25.57 kg-28.73 kg | 342 (20.0%) |
| Q5 | ≥ 44.56 kg | 184 (20.0%) |  | ≥ 28.73 kg | 343 (20.0%) |
| **Cohort study** |  |  |  |  |  |
| Q1 | < 32.01 kg | 43 (20.2%) |  | < 20.50 kg | 106 (20.0%) |
| Q2 | 32.01 kg-36.25 kg | 43 (20.2%) |  | 20.50 kg-23.40 kg | 107 (20.2%) |
| Q3 | 36.25 kg-41.21 kg | 42 (19.7%) |  | 23.40 kg-26.00 kg | 106 (20.0%) |
| Q4 | 41.21 kg-45.21 kg | 42 (19.7%) |  | 26.00 kg-29.21 kg | 105 (19.8%) |
| Q5 | ≥ 45.21 kg | 43 (20.2%) |  | ≥ 29.21 kg | 106 (20.0%) |

HGS, handgrip strength; Q1-Q5, First quintile of HGS - Fifth quintile of HGS; Ref., reference

| **Table S5. Association between HGS and MCI prevalence in male and female ( hierarchical modeling)** | | | | |
| --- | --- | --- | --- | --- |
| **Model** | | **Per 5kg HGS decreased, OR(95% CI)** | ***z*** | ***P*** |
| **Male** | |  |  |  |
|  | **Model 1** | 1.37 (1.26-1.50) | 6.973 | **<0.001** |
|  | **Model 2** | 1.21 (1.09-1.35) | 3.467 | **0.001** |
|  | **Model 3** | 1.19 (1.06-1.33) | 2.948 | **0.003** |
| **Female** | |  |  |  |
|  | **Model 1** | 1.38 (1.25-1.52) | 6.472 | **<0.001** |
|  | **Model 2** | 1.10 (0.98-1.23) | 1.654 | 0.098 |
|  | **Model 3** | 1.10 (0.98-1.23) | 1.587 | 0.113 |

**Model 1:** unadjusted;

**Model 2:** adjusted for age;

**Model 3:** Based on model 2, adjusted for ethnicity, education, marital status, income, BMI, smoking, drinking, physical labor level, hypertension, diabetes, dyslipidemia and coronary heart disease；

OR, odds ratio; CI, confidence interval; MCI, mild cognitive impairment; HGS, handgrip strength

| **Table S6. Association between quintile of HGS and MCI prevalence stratified by age group** | | | | | | | |
| --- | --- | --- | --- | --- | --- | --- | --- |
| **HGS** | **35-60 years** | | |  | **≥60 years** | | |
|  | **OR (95% CI)** | ***P*** | ***P* _for trend_ *^a^*** |  | **OR (95% CI)** | ***P*** | ***P* _for trend_ *^a^*** |
| **Male** |  |  |  |  |  |  |  |
| **Q5** | 1.00 (Ref.) |  | 0.002 |  | 1 (Ref.) |  | 0.1 |
| **Q4** | 1.22 (0.67-2.22) | 0.508 |  |  | 1.60 (0.60-4.33) | 0.345 |  |
| **Q3** | 1.62 (0.87-3.00) | 0.126 |  |  | 1.92 (0.76-4.94) | 0.167 |  |
| **Q2** | 2.02 (1.01-4.04) | 0.046 |  |  | 1.04 (0.42-2.59) | 0.933 |  |
| **Q1** | 4.86 (1.81-13.73) | 0.002 |  |  | 2.36 (0.95-6.00) | 0.065 |  |
| **Female** |  |  |  |  |  |  |  |
| **Q5** | 1.00 (Ref.) |  | 0.236 |  | 1.00 (Ref.) |  | 0.099 |
| **Q4** | 1.26 (0.85-1.88) | 0.249 |  |  | 1.46 (0.63-3.44) | 0.383 |  |
| **Q3** | 1.10 (0.72-1.67) | 0.659 |  |  | 1.08 (0.48-2.46) | 0.859 |  |
| **Q2** | 0.88 (0.56-1.39) | 0.599 |  |  | 0.88 (0.40-1.99) | 0.763 |  |
| **Q1** | 1.79 (1.08-2.99) | 0.025 |  |  | 1.73 (0.80-3.84) | 0.169 |  |

Models adjusted for age, ethnicity, education, marital status, income, BMI, smoking, drinking, physical labor level, hypertension, diabetes, dyslipidemia and coronary heart disease;
a): Tests for trend are based on variables containing median values for each quintile.

OR, odds ratio; CI, confidence interval; MCI, mild cognitive impairment; HGS, handgrip strength; Q1-Q5, First quintile of HGS - Fifth quintile of HGS; Ref., reference

| **Table S7. Association between HGS and incident MCI in male and female (hierarchical modeling)** | | | | |
| --- | --- | --- | --- | --- |
| **Model** | | **Per 5kg HGS decreased, OR(95% CI)** | ***z*** | ***P*** |
| **Male** | |  |  |  |
|  | **Model 1** | 1.16 (0.92-1.49) | 1.21 | 0.225 |
|  | **Model 2** | 0.96 (0.71-1.29) | -0.29 | 0.771 |
|  | **Model 3** | 0.90 (0.64-1.26) | -0.63 | 0.528 |
| **Female** | |  |  |  |
|  | **Model 1** | 1.61 (1.28-2.05) | 4.04 | <0.001 |
|  | **Model 2** | 1.39 (1.07-1.80) | 2.49 | 0.013 |
|  | **Model 3** | 1.45 (1.11-1.92) | 2.69 | 0.007 |

**Model 1:** Adjusted for baseline MoCA-BC;

**Model 2:** Based on model 1, adjusted for age;

**Model 3:** Based on model 2, adjusted for ethnicity, education, income, BMI, smoking, drinking, physical labor level, hypertension, diabetes, dyslipidemia and coronary heart disease；

OR, odds ratio; CI, confidence interval; MCI, mild cognitive impairment; HGS, handgrip strength

| **Table S8.** **Association between HGS and incident** **MCI in male and female, using sole MoCA scale to diagnose MCI( hierarchical modeling)** | | | | |
| --- | --- | --- | --- | --- |
| **Model** | | **Per 5kg HGS decreased, OR(95% CI)** | ***z*** | ***P*** |
| **Male** | |  |  |  |
|  | **Model 1** | 1.18 (0.98-1.45) | 1.693 | 0.090 |
|  | **Model 2** | 1.01 (0.80-1.28) | 0.098 | 0.922 |
|  | **Model 3** | 1.01 (0.77-1.33) | 0.076 | 0.939 |
| **Female** | |  |  |  |
|  | **Model 1** | 1.86 (1.51-2.31) | 5.71 | <0.001 |
|  | **Model 2** | 1.37 (1.08-1.74) | 2.578 | 0.010 |
|  | **Model 3** | 1.40 (1.09-1.80) | 2.608 | 0.009 |

**Model 1:** Adjusted for baseline MoCA-BC;

**Model 2:** Based on model 1, adjusted for age;

**Model 3:** Based on model 2, adjusted for ethnicity, education, income, BMI, smoking, drinking, physical labor level, hypertension, diabetes, dyslipidemia and coronary heart disease；

OR, odds ratio; CI, confidence interval; MCI, mild cognitive impairment; HGS, handgrip strength

| **Table S9. Association between quintile of HGS and incident MCI in male and female, using sole MoCA scale to diagnose MCI (sensitivity analysis)** | | | |
| --- | --- | --- | --- |
| **HGS** | **OR (95% CI)** | ***P*** | ***P* _for trend_ *^a^*** |
| **Male** |  |  |  |
| **Q5** | 1.00 (Ref.) |  | 0.491 |
| **Q4** | 0.95 (0.34-2.63) | 0.922 |  |
| **Q3** | 1.47 (0.49-4.53) | 0.493 |  |
| **Q2** | 1.46 (0.46-4.76) | 0.521 |  |
| **Q1** | 1.35 (0.37-4.99) | 0.647 |  |
| **Female** |  |  |  |
| **Q5** | 1.00 (Ref.) |  | 0.031 |
| **Q4** | 1.10 (0.47-2.59) | 0.826 |  |
| **Q3** | 2.05 (0.96-4.51) | 0.069 |  |
| **Q2** | 1.44 (0.66-3.19) | 0.360 |  |
| **Q1** | 2.56 (1.12-5.99) | 0.027 |  |

Adjusted: age, baseline MOCA-BC score, ethnicity, education, income, BMI, smoking, drinking, physical labor level, hypertension, diabetes, dyslipidemia and coronary heart disease. **OR**, odds ratio; **MCI**, mild cognitive impairment; **HGS**, handgrip strength (HGS was divided into quintiles separately for males and females); Q1-Q5, First quintile of HGS - Fifth quintile of HGS;

**a):** Test for trend based on variables containing the median value for each quintile.

| **Table S10. Association between quintile of HGS and incident MCI in participants ≥ 50 years** | | | |
| --- | --- | --- | --- |
| **HGS** | **OR (95% CI)** | ***P*** | ***P* _for trend_** |
| **Male** |  |  |  |
| Per 5kg HGS decreased | 0.92 (0.64-1.32) | 0.640 |  |
| Q5 | 1.00 (Ref.) |  | 0.665 |
| Q4 | 0.57 (0.10-3.12) | 0.515 |  |
| Q3 | 0.56 (0.10-3.27) | 0.518 |  |
| Q2 | 0.68 (0.14-3.46) | 0.638 |  |
| Q1 | 0.51 (0.09-3.02) | 0.448 |  |
| **Female** |  |  |  |
| Per 5kg HGS decreased | 1.41 (1.06-1.89) | **0.018** |  |
| Q5 | 1.00 (Ref.) |  | 0.110 |
| Q4 | 3.33 (1.00-13.23) | 0.062 |  |
| Q3 | 2.51 (0.79-9.73) | 0.144 |  |
| Q2 | 4.50 (1.49-16.97) | **0.013** |  |
| Q1 | 3.84 (1.25-14.65) | **0.029** |  |

Models adjusted for age, ethnicity, education, income, BMI, smoking, drinking, physical labor level, hypertension, diabetes, dyslipidemia and coronary heart disease;

OR, odds ratio; CI, confidence interval; MCI, mild cognitive impairment; HGS, handgrip strength; Q1-Q5, First quintile of HGS - Fifth quintile of HGS; Ref., reference
